# Monitoring the Long-Term Effectiveness of Miltefosine in Indian Post-Kala-Azar Dermal Leishmaniasis

**DOI:** 10.4269/ajtmh.23-0197

**Published:** 2024-03-05

**Authors:** Sutopa Roy, Srija Moulik, Madhurima Roy, Manab K. Ghosh, Surya Jyati Chaudhuri, Dhruv K. Pandey, Saurabh Jain, Daniel Argaw Dagne, Mitali Chatterjee

**Affiliations:** ^1^ Department of Pharmacology, Institute of Postgraduate Medical Education and Research, Kolkata, India;; ^2^ Department of Tropical Medicine, School of Tropical Medicine, Kolkata, India;; ^3^ Department of Microbiology, Sarat Chandra Chattopadhyay Government Medical College and Hospital, Howrah, India;; ^4^ Kala-Azar Elimination Programme, World Health Organization Country Office, New Delhi, India;; ^5^ Department of Control of Neglected Tropical Diseases, World Health Organization, Geneva, Switzerland

## Abstract

Post-kala-azar dermal leishmaniasis (PKDL), the dermal sequel to visceral leishmaniasis (VL), is characterized by hypopigmented macules (macular) and/or papules and nodules (polymorphic). Post-kala-azar dermal leishmaniasis plays a significant role in disease transmission, emphasizing the need for monitoring chemotherapeutic effectiveness. Accordingly, this study aimed to quantify the parasite burden in PKDL patients after treatment with miltefosine by a quantitative polymerase chain reaction (qPCR). A *Leishmania* kinetoplastid gene-targeted qPCR was undertaken using DNA from skin biopsy specimens of patients with PKDL at three time points, i.e., at disease presentation (week 0, *n =* 157, group 1), upon completion of treatment (week 12, *n =* 39, group 2), and at any time point 6 months after completion of treatment (week ≥36, *n =* 54, group 3). A cycle threshold (*Ct*) <30 was considered the cutoff for positivity, and load was quantified as the number of parasites/µg genomic DNA (gDNA); cure was considered when samples had a *Ct* >30. The parasite load at disease presentation (group 1) was 10,769 (1,339–80,441)/µg gDNA (median [interquartile range]). In groups 2 and 3, qPCR results were negative in 35/39 cases (89.7%) and 48/54 cases (88.8%), respectively. In the 10/93 (10.8%) qPCR-positive cases, the parasite burdens in groups 2 and 3 were 2,420 (1,205–5,661)/µg gDNA and 22,195 (5,524–100,106)/µg gDNA, respectively. Serial monitoring was undertaken in 45 randomly selected cases that had completed treatment; all cases in groups 2 or 3 had a *Ct* >30, indicating cure. Overall, qPCR confirmed an 89.2% cure (as 83/93 cases showed parasite clearance), and the persistent qPCR positivity was attributed to nonadherence to treatment or unresponsiveness to miltefosine and remains to be investigated.

## INTRODUCTION

Leishmaniasis is a neglected tropical disease (NTD) caused by the digenetic parasite *Leishmania* and includes visceral leishmaniasis (VL), which can be fatal if left untreated. Visceral leishmaniasis has an immense global burden, with an estimated 50,000–90,000 new cases occurring worldwide annually.^1^ Visceral leishmaniasis has a dermal sequel, post-kala-azar dermal leishmaniasis (PKDL), which manifests in Southeast Asia in 2.5–20% and in East Africa (mainly Sudan) in up to 60% of patients during or after cure from VL.^2^^–^^4^ Post-kala-azar dermal leishmaniasis in Southeast Asia presents as a combination of macular, papular, and/or nodular lesions (polymorphic PKDL) or only hypomelanotic lesions (macular PKDL), whereas in Sudan, PKDL appears mainly as papulonodular or plaque-like lesions, with a 80% self-healing rate within 6 months to 1 year of their occurrence.^4^^–^^6^ In Southeast Asia, self-healing is rarely observed, and treatment is consistently required for all PKDL cases.^6^ In view of VL trends in Southeast Asia, with an upsurge appearing every 10 to 15 years, a strong contender driving this cyclical pattern is PKDL, which usually occurs in patients 2–10 years after apparent cure from VL.^7^ Therefore, as PKDL cases are considered the likeliest cause for disease transmission, their early identification and effective treatment are crucial.

In 2005, the Kala-Azar Elimination Program (KAEP) was initiated in accordance with a memorandum of understanding signed between India, Bangladesh, and Nepal, with the goal of reducing the annual incidence of VL to less than one case per 10,000 population at a district (Nepal) or subdistrict/upazila level in India and Bangladesh, respectively.^8^ During the early phase of the elimination program, the focus was on surveillance, diagnosis, and treatment of VL, whereas monitoring of PKDL cases was not accorded the same level of priority. However, since 2012, monitoring saw an impetus, as it was reinforced in the national road map for KAEP (August 2014), with adequate emphasis being placed on active surveillance of PKDL.^9^ Factors that contributed to the early detection and dramatic reduction of VL cases in Southeast Asia include the development and wider use of diagnostic tests, especially the rk39 immunochromatographic test, availability of orally administrable miltefosine, and later, a single dose of liposomal amphotericin B (LAmB).^10^^,^^11^ However, the management of PKDL is still challenged by the lack of a user-friendly definitive diagnostic tool applicable at primary health care systems, coupled with the necessity for prolonged treatment.

Molecular diagnostic tools are emerging and include polymerase chain reaction (PCR), loop-mediated isothermal amplification, and recombinase polymerase assay-based approaches using slit-skin smears and/or skin biopsy specimens.^12^^,^^13^ An insurmountable challenge is the detection of Leishman-Donovan (LD) bodies in a substantial proportion of macular cases, and in such instances, negative skin smears do not rule out PKDL.^12^ Following active surveillance, macular cases have been identified as representing up to 50% of the PKDL population^14^^,^^15^ and in Bangladesh up to 90% of PKDL cases.^16^ Although miltefosine has been recommended for PKDL for 12 weeks based on small clinical trials,^17^^,^^18^ these studies did not quantify the parasite burden. Accordingly, this study was undertaken to assess the effectiveness of miltefosine by quantifying the parasite burden at different points of disease presentation, namely, at week zero and after treatment both on a short-term basis (week 12) and at later time points (on follow-up, week >36).

## MATERIALS AND METHODS

### Diagnostic procedures and treatment.

Suspected PKDL patients were recruited based on the presence of macular, papular, and/or nodular lesions, history of VL, and rk39 immunochromatographic strip test positivity according to the accelerated plan for kala-azar elimination program guidelines.^19^ Treated cases of PKDL were recruited based on a prior diagnosis of PKDL. A 4-mm punch biopsy specimen was taken from the lesional site of all PKDL cases (usually a noncosmetic area) along with 1 mL of heparinized blood.

Post-kala-azar dermal leishmaniasis patients were treated with miltefosine (50 mg orally twice daily if body weight >25 kg or 50 mg orally once daily if body weight <25 kg) according to recommended guidelines.^8^

### Study population and design.

Post-kala-azar dermal leishmaniasis patients recruited for this study (*N =* 205) included patients recruited by passive surveillance (*n =* 54, 2007–April 2022) from the outpatient departments of the School of Tropical Medicine/Calcutta Medical College/Institute of Post Graduate Medical Education and Research and S.S.K.M. Hospital, Kolkata, West Bengal, whereas the remaining PKDL patients were recruited by active surveillance (*n =* 151, 2015–April 2022) from Jharkhand (Dumka, Pakur, Sahibganj, and Godda, *n =* 40) and West Bengal (Malda, Murshidabad, Dakshin Dinajpur, and Birbhum, *n =* 111), after their clinical examination in medical camps.

**Figure 1.**
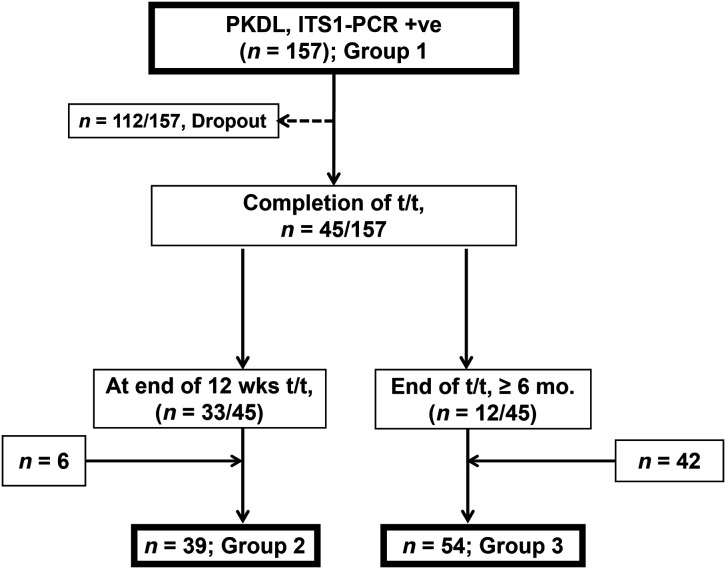
Schematic representation of naïve and miltefosine-treated cases of post-kala-azar dermal leishmaniasis (PKDL) based on the time point. ITS1-PCR +ve = internal transcribed spacer 1-polymerase chain reaction positive; mo. = months; t/t = treatment; wks = weeks.

The study population presented with dermal lesions (papules, nodules, and/or macules), and the majority reported a history of VL (some individuals failed to recall) and tested positive on the rk39 strip test (using peripheral blood) (Table 1). Potential sources of bias included failure to accurately recall precise information (e.g., drug intake, date of start and completion of treatment, and also time of lesion appearance and clearance). The diagnosis was confirmed by PCR using internal transcribed sequence 1 (ITS-1 PCR) using skin biopsy specimens.^20^ These 205 cases were collected at three time points (Figure 1): 1) at disease presentation (group 1, *n* = 157, week 0); 2) after completion of 12 weeks of treatment with miltefosine (group 2, *n* = 39, week 12) (this group included 33 cases that were followed up from group 1, along with an additional 6 cases that reported only upon completion of 12 weeks of miltefosine treatment) (Figure 1); and 3) at least 6 months after completion of treatment (group 3, *n* = 54, week >36) (this group included 12 follow-up cases [from group 1] along with 42 cases who presented after 6 months following completion of treatment only (*n =* 54) (Figure 1). In sum, this study provided parasite burden from 250 skin biopsy specimens (group 1, *n =* 157; group 2, *n =* 39; and group 3, *n =* 54) sourced from 205 confirmed PKDL cases (group 1, *n =* 157; group 2, *n =* 6; and group 3, *n =* 42) (Figure 1).

**Table 1:** Study population of PKDL cases from districts of West Bengal and Jharkhand where kala-azar is endemic*

Clinical features	Group 1, Week 0 (*n* = 157)	Group 2, Week 12 (*n* = 6)^‡^	Group 3, Week ≥36 (*n* = 42)^§^
Age in years^†^	22 (16.0–32.0)	19.50 (16.0–29.5)	23 (16.0–32.5)
Sex (males:females)	99:58 (1.7:1)	2:4 (1:2)	27:15 (1.8:1)
History of VL	92.36%	100%	97.6%
Lesion type (polymorphic:macular)	85:72 (1.18:1)	5:1	15:27 (1:1.8)
Lag period^‖^ in years^†^	4.0 (2.0–7.0)	7.0 (2.2–14.0)	8.0 (4.5–11.2)
PKDL duration^¶^ in years^†^	2 (1–5)	1 (1–2)	2 (1–3)
Parasite load (parasites/µg gDNA)^†^	10,769 (1,339–80,441)	1,525 (1–3,670)	1 (1–1)

gDNA = genomic DNA; PKDL = post-kala-azar dermal leishmaniasis; t/t = treatment; VL = visceral leishmaniasis.

* The study population included 205 patients with PKDL that were subdivided into three groups depending on the time of presentation, group 1 being at disease presentation, group 2 being after completion of 12 weeks of treatment, and group 3 being at any time point at least 6 months after the end of treatment. The skin biopsy specimens were collected as described in Materials and Methods.

^†^ All values are in median (interquartile range).

^‡^ Thirty-three patients entered the study in group 1.

^§^ Twelve patients entered the study in group 1.

^^‖^^ The lag period is the interval between cure of VL and onset of PKDL.

^¶^ PKDL duration indicates the time from appearance of lesions to presentation at the medical camp.

Depending on the distribution of lesions, the PKDL cases (*n =* 205) included individuals with polymorphic features (*n =* 105), while others had only hypomelanotic patches and were considered cases of macular PKDL (*n =* 100) (Table 1). Individuals recruited for the study had no comorbidities; additionally, as miltefosine is teratogenic, pregnant and lactating women were excluded.

### Determination of parasite load by quantitative real-time PCR (qPCR).

For quantification of the parasite load, a standard curve was generated by serially diluting DNA isolated from 1 × 10^7^ *Leishmania donovani* parasites (ranging from 1 × 10^6^ to 1 × 10^1^) and proceeding with real-time qPCR using minicircle kinetoplastid DNA-specific primers (116 bp; forward, 5′-CCTATTTTACACCAACCCCCAGT-3′, and reverse, 5′-GGGTAGGGGCGTTCTGCGAAA-3′). Briefly, DNA (50 ng/µL) was added to a 19-µL reaction mixture containing PowerUp SYBR green master mix and a 400 nM concentration of each primer as previously described.^20^ DNA from *Leishmania donovani* strain MHOM/IN/80/DD8 served as the positive control, whereas a reaction mixture containing water instead of the DNA template served as the non-template control. The parasite load of lesional biopsy specimens was extrapolated from the generated standard curve and is given as the number of parasites/µg genomic DNA (gDNA). In previous studies, healthy controls consistently gave a cycle threshold (*Ct*) value >30; accordingly, a *Ct* cutoff value ≥30 was considered negative, indicating parasite burden clearance/cure. The parasite load of these negative samples were accorded an arbitrary value of 1.^20^

All reagents used in the study were from Sigma-Aldrich (St. Louis, MO), except for the QIAamp DNA mini kit, which was from Qiagen (Hilden, Germany), the Power SYBR green master mix, which was from Applied Biosystems (Grand Island, NY), primers, which were from Integrated DNA Technologies (IDT, Coralville, IA), and the rK39 immunochromatographic test strips, which were from InBios International (Seattle, WA).

## STATISTICAL ANALYSES

All data are expressed as the median (interquartile range [IQR]). Data normality was checked using the D’Agostino and Pearson omnibus normality test. Comparison between the nonparametric data of two groups was undertaken using the Mann-Whitney test, whereas three groups were analyzed by the Kruskal-Wallis test, followed by the Dunn multiple comparison test using GraphPad Prism software version 8.0 (GraphPad Software, La Jolla, CA); *P <*0.05 was considered significant (95% CI).

## RESULTS

### Study population.

The study included 250 samples from 205 patients who showed no gender bias and were broadly subdivided into: 1) group 1 (*n =* 157), which presented at diagnosis (week 0); 2) group 2, which included 39 cases that presented after completion of treatment (week 12); and 3) group 3, which included 54 patients who presented at least 6 months after completion of treatment with miltefosine (week ≥36) (Table 1, Figure 1). More than 96% of the patients reported a history of VL, and the lesional distributions of polymorphic and macular cases were comparable, as were the lag periods, though they showed substantial variation (Table 1). Among group 1 patients, 71.3% (112 of 157) did not report during scheduled follow-up. The high dropout rate was not associated with confounders at the time of diagnosis, as recruited patients did not have comorbidities.

**Figure 2.**
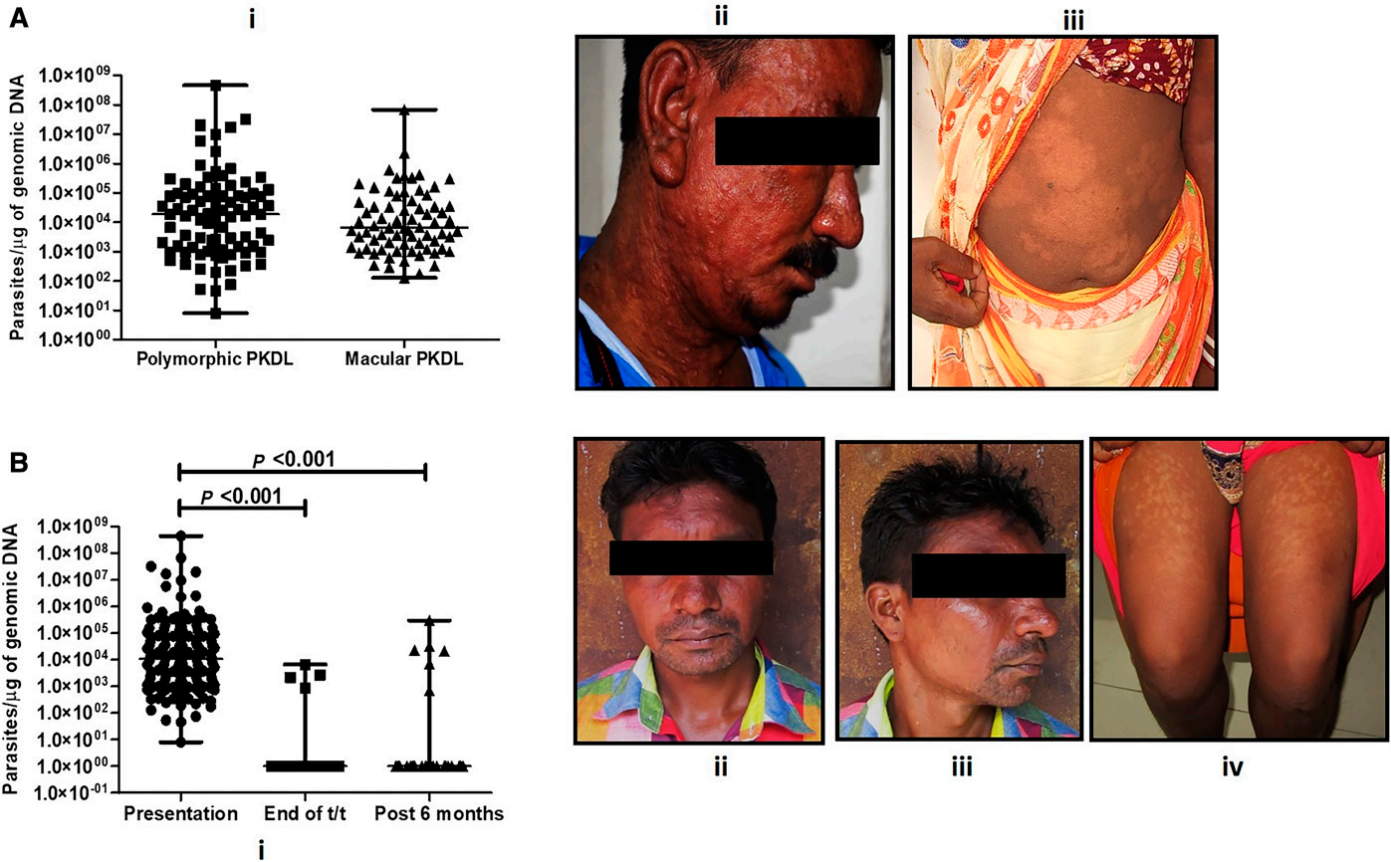
(**A**) Status of parasite load in patients with post-kala-azar dermal leishmaniasis (PKDL). (i) Scatter plots showing the parasite load in PKDL patients with polymorphic (■; *n* = 85) or macular (▲; *n* = 72) lesions as described in Materials and Methods. Representative images of PKDL patients with polymorphic (ii) or macular (iii) lesions. (**B**) Effect of miltefosine on the parasite load of patients with PKDL. (i) Scatter plots indicating parasite load at disease presentation (●; *n* = 157) at the end of 12 weeks treatment with miltefosine (■; *n* = 39) and at 6 months posttreatment (▲; *n* = 65). Representative images of PKDL patients with persisting polymorphic (ii and iii) or macular (iv) lesions after 2 years and after 6 months, respectively.

In group 1, the parasite load was 10,769 (1,339–80,441)/µg gDNA median (IQR), which when subdivided on a lesional basis indicated that patients with the polymorphic form (*n =* 85) exhibited a 2.84 fold-higher parasite burden than those with the macular type (*n =* 72), being 18,620 (1,266–93,934) versus 6,548 (1,306–44,514)/µg gDNA, respectively, but the difference was not statistically significant (Figure 2A). On completion of 12 weeks of treatment with miltefosine, 35/39 (89.7%) PKDL patients (group 2) irrespective of their lesional variant demonstrated a significant decrease in parasite load, as it decreased to 1 (1–1)/µg gDNA (95% CI; *P <*0.001); the parasite load in the 4/39 (10.2%) qPCR-positive patients was 2,420 (1,205–5,661)/µg gDNA (Figure 2B), and the ratio of patients with the polymorphic form to the macular form was 3:1. In the case of PKDL patients who reported at least 6 months after completion of treatment, i.e., group 3, 48/54 (88.8%) had cleared their parasite burdens, the load being 1 (1–1)/µg gDNA (95% CI; *P <*0.001), whereas in 6/54 (11.2%) cases, qPCR results were positive, and the parasite load persisted, being 22,195 (5,524–100,106)/µg gDNA (Figure 2B). These six cases included four with the polymorphic form of disease, who presented between 6 and 12 months (*n =* 2) or >2 years after treatment (*n* = 2); the remaining two cases had the macular type and presented >2 years after completion of treatment. Furthermore, when patients were subclassified based on their recruitment through active or passive surveillance, it was observed that all 10 qPCR-positive patients were recruited through active surveillance.

In terms of resolution of clinical features, the patients in groups 2 and 3 were analyzed. In sum, 83/93 patients (group 2, *n =* 35/39; group 3, *n* = 48/54), having an arbitrary parasite load score of 1, included 44 polymorphic and 39 macular cases of PKDL; among them, 52/83 (63.0%) continued to show lesions as assessed by the patient himself/herself. In group 2, the ratio of patients with the polymorphic form of the disease to the macular type was 25:10; among the patients with the polymorphic form, 16/25 (64%) continued to show lesions, whereas among the those with the macular form, lesions persisted in 7/10 (70%). In group 3, among the 48 patients, the ratio of polymorphic cases to macular cases was 19:29; in the polymorphic group, 18/19 (95%) continued to show lesions, whereas among the 29 patients with the macular form, lesions persisted in 11/29 (38%). Among the qPCR-positive cases, lesions persisted in 9/10 (macular, *n =* 5; polymorphic, *n =* 4).

### Longitudinal monitoring of parasite load of PKDL patients treated with miltefosine.

**Figure 3.**
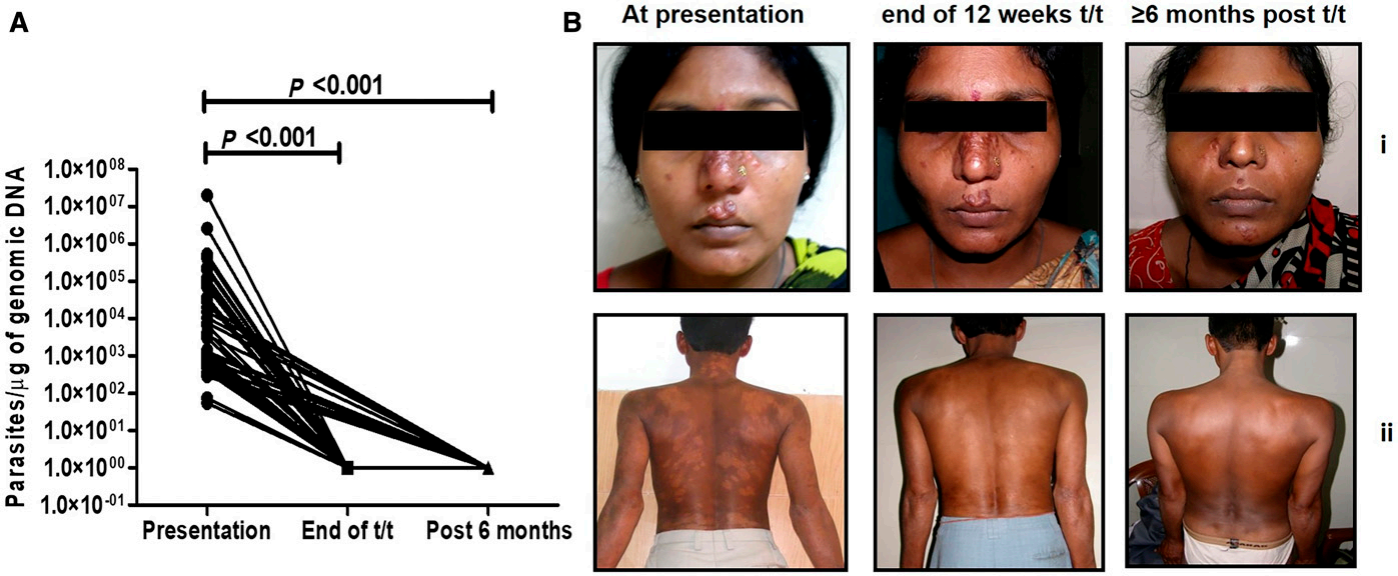
Longitudinal monitoring of the effectiveness of miltefosine in post-kala-azar dermal leishmaniasis (PKDL). (**A**) Before-and-after plots indicating parasite load at disease presentation (●; week 0), at the end of 12 weeks of treatment (t/t) with miltefosine (■; week 12) and/or at ≥6 months posttreatment (▲; week > 36); *P <*0.001 as compared with presentation. (**B**) Representative clinical profiles of longitudinally monitored patients with polymorphic PKDL (i) or macular PKDL (ii), at disease presentation, at the end of 12 weeks treatment with miltefosine, and at ≥6 months posttreatment.

Longitudinal monitoring (i.e., availability of a biopsy specimen for at least two time points from the same patient) was possible for 45 patients with PKDL (2009–2016), with a median age of 25.0 (16.0–37.5) years, a ratio of males to females of 32:13, and a ratio of polymorphic to macular cases of 31:14. The lag period, i.e., gap between cure from VL and appearance of PKDL skin lesions, in this group was 3 (2–6) years and showed no correlation with the parasite load. The parasite load was 1.29-fold higher in cases with the polymorphic form than in cases with the macular type, being 3,499 (778–70,270) and 2,694 (820.8–28,938)/µg gDNA, respectively, and the difference was not statistically significant. In 11 cases, samples were obtained at all three time points, i.e., at weeks 0, 12, and >36. Additionally, there were 33 patients whose samples were available at disease presentation and at the end of 12 weeks of miltefosine treatment, i.e., weeks 0 and 12. In only one case was a sample available at disease presentation and >6 months posttreatment, i.e., at weeks 0 and >36. Irrespective of whether the sample was collected at week 12 or >36, the parasite load was 1 (1–1)/µg gDNA (*P <*0.001, 95% CI) (Figure 3A and B). In terms of clinical features, lesions persisted in 32/45 (71.1%) patients and included: 1) 12 patients who presented at the end of 12 weeks of treatment (polymorphic/macular ratio being 3:9), 2) 19 patients who presented 6 to 11 months posttreatment (polymorphic/macular ratio being 10:9), and 3) one patient with macular PKDL that presented ≥12 months later.

## DISCUSSION

Post-kala-azar dermal leishmaniasis cases as silent mobile disease reservoirs are a major deterrent to the success of the ongoing KAEP, as they can facilitate disease transmission and potentially cause outbreaks of VL.^7^^,^^10^ Therefore, with implementation of active surveillance, a huge disease burden of PKDL has been unearthed in Southeast Asia.^5^^,^^14^ In particular, the proportion of the macular form, especially in females that were previously reported to constitute <10% of cases of PKDL,^12^ has been drastically modified to a male/female ratio of 1.1:1^21^ and is reflected in the study population (Table 1, Figure 1). The macular form is challenging from a diagnostic and therapeutic perspective, as LD bodies are minimally present in the lesions of this form and their detection requires considerable expertise.^12^ This challenge can be resolved only by applying molecular tools such as ITS-1 PCR and qPCR, as endorsed in this study, with the polymorphic form of the disease having a 2.84-times higher parasite load than the macular counterpart (Figure 2A).

In prior xeno-diagnostic studies, irrespective of the lesional type, the parasite burden in PKDL cases ranging from 1,428 to 63,058 parasites/µg gDNA were demonstrated to be capable of transmitting disease.^22^ Therefore, as the parasite burden in both polymorphic and macular cases of PKDL in this study were within this range (Figure 2), they can be considered disease transmitters. Accordingly, if the elimination program is to succeed, it is necessary to curb transmission, and this study reiterated the need for the treating physician and health worker to be sensitized regarding the importance of early detection and prompt treatment of PKDL cases.^23^

Another challenge is that the clinical features of macular PKDL are practically indistinguishable from those of other hypopigmentary dermatoses prevalent in the same geographical area, including leprosy, another neglected tropical disease,^24^ pityriasis versicolor,^25^ and rarely even vitiligo.^26^ Additionally, most hypopigmentary disorders have a considerable component of social rejection, and women who presently comprise almost 50% of PKDL cases^5^ are often reluctant to enter the treatment fold. These issues need to be addressed by implementing a national integrated skin NTD surveillance approach, which may well prove to be an effective sustainable strategy.

Irrespective of the chemotherapeutic approach, the current treatment options available for PKDL are prolonged, and unfortunately, to date, the regimens are empirical and based on maximally tolerated doses rather than the rational and optimized delineation of their pharmacokinetics. The prolonged treatment of PKDL with miltefosine is associated with several adverse effects, such as nausea, vomiting, and, in recent times, ocular damage.^27^ The clinical readout for effectiveness is based on the resolution of lesions, but the major constraint is that despite completion of the 12-week regimen by patients, hypopigmentation persists and repigmentation can occur at least 6 to 12 months later and sometimes even later.^28^ In this study, 52/83 (63.0%) patients showed a reduction in lesions, but because their lesions persisted after completion of treatment, these patients would not have been considered “cured” according to the clinical definition.^19^ This posed a clinical dilemma to the treating physician coupled with the patient’s dissatisfaction, as the loss of pigmentation was their primary motivation to seek treatment (Mitali Chatterjee, personal communication). Therefore, appropriate counseling and the use of molecular tools can obviate this limitation, with quantification of parasite load being an objective parameter of efficacy (Figures 2 and 3). This study sends an important message to the treating physician and health worker on the importance of early detection and prompt treatment of PKDL cases to curb transmission. The target product profile for a point-of-care diagnostic test for dermal leishmaniasis should encourage researchers and developers to provide better diagnostic tests for PKDL.^29^

This study corroborated the long-term effectiveness of miltefosine (Figure 3A and B), but only 45 patients could be serially monitored for at least at two time points, indicating that more studies are needed to confirm the effectiveness of qPCR in monitoring treatment. The persistence of lesions in at least 50% of cases emphasized the need for counseling and guiding the patients, as well as the importance of treatment adherence and follow-up. Furthermore, persistence of lesions even beyond the 6-month period reiterated the importance of long-term follow-up of PKDL patients and is aligned with the current practice of 3 years of follow-up according to the revised treatment card followed by KAEP.^30^ Additionally, 10/93 (11%) patients followed up after miltefosine treatment continued to be qPCR positive (Figure 2B), corroborating studies highlighting the decline in miltefosine efficacy in PKDL.^18^^,^^31^ It is important to pinpoint whether this unresponsiveness is host or parasite mediated; a plausible reason for the decline could be attributed to miltefosine resistance but can be validated only by performing drug sensitivity assays, which are a logistical challenge.^32^ At this point, the possibility of parasite reemergence cannot be ruled out, as Rugani et al. reported the presence of intramacrophage quiescent amastigotes being capable of restoring infection despite their susceptibility to miltefosine.^33^ The lesions of PKDL are generally disseminated, suggesting the systemic nature of the disease. This was confirmed by quantification of the parasite load in three naive polymorphic PKDL cases, wherein biopsy specimens were taken from multiple lesions (papule, nodule, and macule). The parasite distribution was remarkably consistent, irrespective of the type or location of the lesion,^20^ and confirmed the systemic distribution of parasites. Therefore, for monitoring parasite clearance, a biopsy specimen collected from a lesional site is representative of all lesions.

The long-term monitoring of parasite kinetics in PKDL patients treated with a 3-week regimen of LAmB indicated a substantial decrease in the parasite load but was associated with a marked resurgence of parasites 6 months after completion of treatment.^20^ In the same study, 38 PKDL patients who received miltefosine showed a total decline in parasite burden and remained negative even 6 months after treatment.^20^ Pandey et al., in a randomized open label study, assessed the efficacies of LAmB and miltefosine in PKDL patients and reported cure rates of 74.5% and 86.9%, respectively.^34^ In view of the limited armamentarium against VL and PKDL, as well as the unresponsiveness to LAmB and miltefosine, this study has reiterated the importance of appropriate counseling for patients to ensure early diagnosis as well as adherence to treatment and regular follow-up visits. Because the VL burden in Southeast Asia has decreased significantly, the national KAEP should continue to pay special attention to the detection of PKDL and to ascertain the patients’ clinical cure. Major hurdles in loss to follow-up can be resolved by linking the current incentive scheme of wage loss compensation of PKDL patients (USD 50), inclusive of follow-ups. Furthermore, because PKDL occurs after treatment with all antileishmanial agents used in VL, this study highlights the need to follow up all VL cases up to 3 years posttreatment to detect the majority of PKDL cases and is aligned with the proposed target for kala-azar in the new NTD roadmap.^30^ Additionally, evaluations of new and shorter combination therapies, e.g., liposomal amphotericin B plus miltefosine and miltefosine plus paromomycin, are likely to accelerate the elimination drive and, additionally, prevent occurrence of the ocular complications that can accompany prolonged treatment with miltefosine.^27^

The limitations of this study included a high dropout rate after diagnosis, which was attributed to the time period of this study coinciding with the COVID-19 pandemic and the follow-up time point being restricted to 36 months. Additionally, the established poor health-seeking behavior may also have a contributory role.^35^ However, in the absence of any documentary evidence, it remains an open-ended yet pertinent question as to whether the patients showed poor medication adherence or were unresponsive/resistant to miltefosine. Furthermore, unlike earlier studies where the disappearance of lesions at a 12-month follow-up was considered as the criterion of parasitological and clinical cure, this assessment was not achievable in this study as majority of patients failed to report. This can be attributed to multiple reasons: e.g., economic, as the disease occurs in the poorest segment of society; social, owing to the potential stigmatizing clinical features of PKDL, especially hypopigmentation; and perhaps most importantly, the nonfatal nature of the disease.^35^ Taken together, this study suggests that molecular tools be considered for diagnosis and for monitoring treatment efficacy, especially as antibody-based positivity can be attributed to a past infection with VL.^36^ Accordingly, cost, feasibility, and other operational aspects could be assessed to possibly leverage the use of existing human resources and equipment, e.g., thermocyclers that were installed for the diagnosis of COVID-19 at a health facility level.
